# The story behind the synthesis: writing an effective introduction to your scoping review

**DOI:** 10.1007/s40037-022-00719-7

**Published:** 2022-08-12

**Authors:** Lorelei Lingard, Heather Colquhoun

**Affiliations:** 1 Centre for Education Research & Innovation, and Department of Medicine, Schulich School of Medicine & Dentistry and Faculty of Education, Western University, London, Canada; 2 Department of Occupational Science & Occupational Therapy, University of Toronto, Toronto, Canada

In the Writer’s Craft section we offer simple tips to improve your writing in one of three areas: Energy, Clarity and Persuasiveness. Each entry focuses on a key writing feature or strategy, illustrates how it commonly goes wrong, teaches the grammatical underpinnings necessary to understand it and offers suggestions to wield it effectively. We encourage readers to share comments on or suggestions for this section on Twitter, using the hashtag: #how’syourwriting?

In a recent writing workshop, a participant was applying the “mapping the gap” heuristic in the introduction of his paper. Mapping the gap is a strategy for writing a succinct, compelling literature review in a paper’s Introduction: the writer briefly and selectively summarizes what’s known in order to outline the white space—the gap—that the research fills [1]. But this writer had done a scoping review, and he was struggling to make the heuristic fit. “*How do I summarize what is already known,*” he said, “*without giving away what my scoping review found? The gap is one of my results, isn’t it*?”

Good question. And for anyone writing or giving feedback on scoping reviews, a rather pressing one. This form of knowledge synthesis is proliferating in health research generally and health professions education (HPE) research specifically, and a recent review suggests that published scoping review manuscripts in HPE often lack a strong introductory rationale for the work [2]. This may be in part because guidance for writers regarding this section of a scoping review is sorely lacking. The problem isn’t a lack of frameworks or guidelines for conducting and reporting scoping reviews: these are available in abundance, with ongoing updates and refinements [3, 4]. But in all cases the emphasis is predominantly on methods: the idea of the scoping review as a story to be told is missing.

Two main sources of scoping review guidance illustrate this point. The Joanna Briggs Institute (JBI) guidelines for designing scoping reviews [4] give more attention to title than introduction: the advice is mainly that the latter “should be comprehensive and should cover the main elements of the topic, important definitions, and the existing knowledge in the field” (11.3.4). The Preferred Reporting Items for Systematic reviews and Meta-Analyses extension for Scoping Reviews (PRISMA-ScR) checklist [5] focuses significantly more attention on Methods and Results than on Introductory framing. Writers find only two items to guide them: we are to “describe the rationale for the review in the context of what is already known… and explain why the review questions/objectives lend themselves to a scoping review approach”; and we should provide “an explicit statement of the questions and objectives being addressed with reference to their key elements (e.g., population or participants, concepts, and context)” (*p* 469). However, as my workshop participant pointed out, the devil is in the details. Where’s the line between describing a rationale in “context of what is already known” and giving away your results, or even negating the value of doing a scoping review in the first place? How much, and which, literature needs to be introduced to establish the “key elements… population or participants, concepts, and context” for the review questions and objectives? In a nutshell, how does the author set up the story of a scoping review? To date, the scoping review guidance literature privileges study—and ignores story. But every good manuscript needs both.

In this Writer’s Craft, we offer writers advice for crafting a clear, compelling story in the Introduction of their scoping review paper. This advice is relevant for any scoping review, thus our illustrations come from a variety of fields.

## Articulate the problem/gap/hook

Scoping reviews are a synthesis of knowledge. Therefore, the introduction’s main purpose is to offer an argument for *why we need a synthesis* of the knowledge on a particular concept. The Problem-Gap-Hook heuristic [6] can help efficiently lay out this argument.

Let’s begin with the Gap, as it is the same for every scoping review: the lack of synthesis of existing literature. This should be signaled clearly with language such as “Amid such debates, the scholarly community **lacks** a large-scale, systematic overview of the [arts & humanities] literature” [7] (*p* 1213) or “there is **little evidence** on how the Bangladeshi community gain access to diabetes-related information and services” [8] (*p* 157), or “one area of medical education research that has **not yet been systematically examined** is family medicine” [9](*p* 1). It is possible to leave the Gap unstated; readers will be able to infer it as it is similar across scoping reviews. However, making it explicit offers the opportunity to characterize it precisely as Maggio et al. [2] do in their scoping review of HPE scoping reviews (*p* 690):

the extant research on scoping reviews provides **limited information about their nature, including how they are conducted, if they are funded, or why medical educators decide to undertake this type of knowledge synthesis in the first place. This lack of direct insight** makes it difficult to know where the field stands and may hamper attempts to take evidence-informed steps to improve the conduct, reporting and utility of scoping reviews in medical education.

Explicitly naming and characterizing the gap is particularly important in a scoping review of scoping reviews, to convince the reader that, amid a sea of reviews, we need another. As scoping reviews proliferate, so too will scoping reviews of scoping reviews, making a strongly characterized gap an essential ingredient of these stories.

While the Gap for a scoping review is invariably some lack of synthesis, this in itself does not justify doing a scoping review. Lots of literature remains unsynthesized; why does it matter in this case? Writers must articulate the Problem that arises because of the lack of synthesis. It is not sufficient to say, for instance, that “Hundreds of knowledge translation (KT) theories exist across a broad range of paradigms including organizational theory, learning theory, and social cognitive theory, but they have yet to be synthesized”. You must make explicit the problem created by an abundance of unsynthesized theoretical KT literature: something like, “Scholars struggle to use KT theory effectively, faced with hundreds of possibilities and no synthesis to guide them”. Look at published scoping reviews to see how other authors express the problem: it might be that the field lacks conceptual consensus, or practices are inconsistent, or controversies remain unresolved, or understanding is inhibited by implicit blind spots, or research is proceeding without programmatic direction. For example, Van Schalkwyk et al. [10] conducted their scoping review of transformative learning as pedagogy because they realized that “understanding of the construct differed amongst us and lacked a clear theoretically grounded comprehension” (*p* 538); Sebok-Syer et al. [11] argued that a scoping review was needed around measuring interdependence because “variability in both terminology and approaches among researchers may contribute to assessment challenges” (*p* 1124); and Young et al. [12] declared that, although it has been much studied, “little consensus exists regarding the definition of clinical reasoning” (*p* 2). Reading critically can help expand your repertoire of phrases for this key part of your Introduction.

The Hook is the ‘so what’ of your scoping review—the statement of why it matters to solve the Problem you’ve identified. The Hook can be expressed either in terms of possibility (e.g., “With a synthesis, we will be able to …”) or caution (e.g., “Without a synthesis, we risk …”). A good scoping review Hook focuses on what the results of the review will be used for, and writers should aim for specific examples of value. Saying that your review will “be useful to a broad audience of educators” is too vague. Be more precise and persuasive. Howell et al. [13] express “**an urgent need** for a guiding taxonomy of core PRO domains and dimensions in cancer” (*p* 77); Gottlieb et al. [14] argue that “**Given the profound impact** of burnout on medicine, understanding imposter syndrome within the context of physicians and physicians in training is **critical**”(*p* 117); Maggio et al. [2] set out to “**identify areas for improvement** in the conduct and reporting of scoping reviews in medical education, **thereby helping to ensure that** those produced are relevant to and practical” (*p* 690); and Young et al. [12] assert that “a careful mapping of the concept of clinical reasoning across professions **is necessary to support** both profession-specific and interprofessional learning, assessment, and research” (*p* 2). A negative hook, with its articulation of problems or negative impacts associated with not having synthesized the literature, may be particularly effective. If we convert Young et al.’s hook to negative, we get something like “without this careful mapping, learning, assessment and research **cannot advance coherently**”. Which do you find more compelling?

## Structure the story

The Problem, Gap and Hook are important, but they are only three of the sentences in your Introduction. What goes in the other sentences, and how should you organize them?

**Fig. 1:**
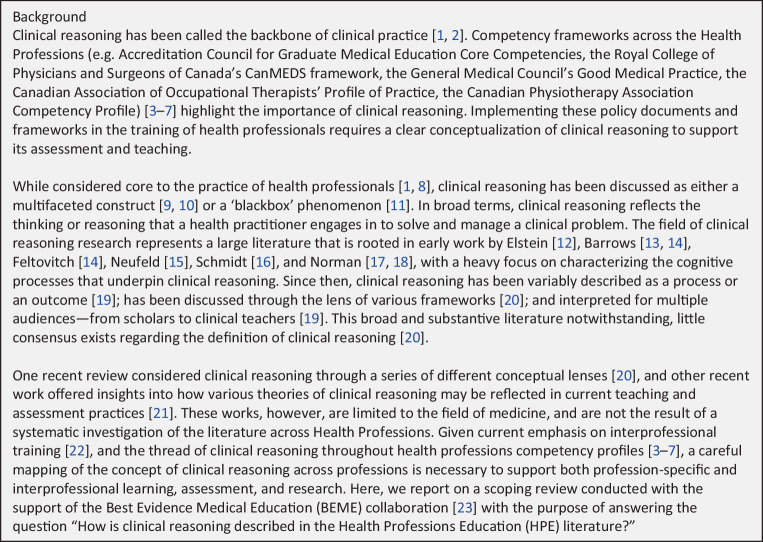
An effective scoping review Introduction, from Young et al. [12]

According to scoping review guidelines [4, 5], the introductory literature review of your paper must do three things: 1) provide relevant details about the “population or participants, concepts, and context” (PCC); 2) establish that there is sufficient literature to warrant a review; and 3) acknowledge any pre-existing reviews and distinguish them from yours. Let’s walk through each of these, unpacking Young et al.’s three-paragraph scoping review introduction as a primary illustration and using additional examples to help you build your Introduction repertoire (Fig. 1).

### Use the PCC framework

According to Peters et al. [15], “use of the PCC mnemonic clearly identifies the focus and context of a review” (p2122). We like to think of it as orienting the reader to the setting and main characters of your story. Young et al. establish the PCC in their first paragraph: they describe the population/participants (health professionals), the main concept (clinical reasoning), and the context (health professions education, including both policy frameworks and teaching/assessment activities). Many scoping review introductions take multiple paragraphs to establish the PCC, starting more generally and then narrowing. This inverted triangle is common in scoping review introductions: for example, O’Brien et al.’s [16] two-paragraph introduction which begins with a paragraph describing the broader context of HIV, followed by a paragraph narrowing in on the concept of rehabilitation in HIV. An inverted triangle introduction doesn’t need to be long: in three paragraphs Maggio et al.’s [2] introduction sketches the rise of scoping reviews in medical education, narrows to scoping review methods and then narrows further to methodological and reporting concerns. By contrast, Alam et al.’s [8] scoping review opens with a protracted inverted triangle which traverses concepts of diabetes, health access, and minority groups before finally coming to its focus in the 10th paragraph of the literature review: British Bangladeshi’s access to diabetes-related healthcare information and services. Particularly if your paper is a conventional length for a health research journal (3000–4000 words), this is a long wait for the appearance of the main character of your scoping review story. Aim for a 3–4 paragraph introduction, use the inverted triangle structure as an organizing logic, and choose stress positions for key sentences (like Young et al.’s problem sentence that concludes the second paragraph).

### Establish that the literature can support a scoping review

There is no magic number of studies that constitutes a threshold for scoping, so the writer must convince the reader that the literature is sufficient. In their second introductory paragraph, Young et al. make this argument. The first sentence notes the “variety” of ways the concept has been discussed; the third sentence characterizes the literature as “large” with an “early” emphasis on “cognitive processes”; the fourth notes that the concept has been approached as both “process” and “outcome”, that it has been variously framed and “interpreted for multiple audiences”. Notice that the detailed content of the literature is not given away: the map is sketched only enough to show its “broad and substantive” contours and set up the problem of “little consensus … regarding the definition of clinical reasoning”. The Young et al. example establishes that there is an abundance of literature for scoping. But that’s not always the case. If you’ve scoped a limited literature, your argument must acknowledge that the literature is limited while explaining why it is still worthwhile to review. For example, O’Brien et al.’s [16] introduction acknowledges that there is only a “small amount of evidence” and that “relatively little research focuses on rehabilitation in HIV care” (*p* 449). Explaining that “this field is still emerging”, they position the review as “an initial step” aimed at “understanding the research priorities” (*p* 449). And, reflecting the limited literature, they also employ key informant consultation to help identify key research priorities and gaps in the field.

### Acknowledge previous reviews

If yours is the first review of the concept with reference to the particular context and participants you’ve outlined, you can simply say so, as Shorey et al. [17] do with their assertion that “there are no existing reviews that have consolidated evidence from studies across all medical faculties” (*p* 767). However, if the literature has already been reviewed, your effort to justify the need for your review needs a bit more attention. Young et al.’s third introductory paragraph recognizes the existence of other reviews and conceptual analyses of clinical reasoning, points out their “limited” focus on medicine, and argues for the need for a synthesis of “literature across Health Professions”. This justification is followed by a positive Hook that **labels what’s distinctive** about their review: “a careful mapping of the concept of clinical reasoning **across professions** is necessary to support both profession-specific and interprofessional learning, assessment, and research.”

How you handle previous reviews in your introduction is particularly important in scoping reviews of scoping reviews. The third paragraph of Maggio et al.’s [2] Introduction makes space for their review by pointing explicitly to “the rise” in scoping reviews in medical education, acknowledging the presence and value of “discipline-specific and cross-disciplinary scoping reviews of scoping reviews”, and arguing that existing reviews are either “several years old” or “focused solely” on one discipline (*p* 690). By asserting that “the multi-disciplinary nature of medical education research” suggests “differences [that] warranted further exploration” (*p* 690), they claim a unique space for their own work amid what the reader may see as a crowded synthesis landscape. Similarly, Howell et al. [13] acknowledge that “our work built on earlier studies, but unlike earlier reviews we used formal methods to gain consensus on core PRO domains and related subdimensions” (*p* 77), and Chan et al. [18] recognize that “there have been some reviews about the use of social media for education” but explain that “none have sought to fully encompass the breadth of how these technologies have affected the full spectrum of education” (*p* 21). As these examples show, the acknowledgement of existing reviews must be accompanied by a clear statement of what your review adds, which should be tied to the problem you’ve outlined.

## Create alignment

The final ingredient of an effective introduction is alignment among the “rationale”, “research question”, and “objectives” of the review [3]. These terms are used variably in scoping reviews, reporting guidelines, and reviews of scoping reviews. For our purposes, we use “rationale” to mean why we’ve done a scoping review—what problem does it address? (This is also sometimes referred to as “purpose” in published scoping reviews.) We use “research question” to mean the specific question guiding the search, and we use “objectives” synonymously with “sub-questions” to represent specific foci of inquiry.

Let’s analyze an example where alignment is achieved. Versteeg et al. [19] briefly summarize the literature and land on this statement of the problem: “Overall, the broad range of terms associated with spaced learning, the multiple definitions and variety of applications used in HPE can hinder the operationalisation of spaced learning” (*p* 206). With this problem in mind, their rationale is “to investigate how spaced learning is defined and applied across HPE contexts”, which they phrase as an overarching question: “How is spaced learning defined and applied in HPE?” (*p* 206). They then articulate “specific research questions: (RQ1A) Which concepts are used to define spaced learning and associated terms? (RQ1B) To what extent do these terms show conceptual overlap? (RQ2) Which theoretical frameworks are used to frame spaced learning? (RQ3) Which spacing formats are utilised in spaced learning research?” (*p* 206) This example is well-aligned: “definition and application of spaced learning” remains consistently in focus, and refinements such as “conceptual overlap” and “theoretical frameworks” are further and logical specifications of the overall focus. This example illustrates Levac et al.’s [3] advice to balance broad research questions with clearly articulated scope of the inquiry and link the rationale to the research questions.

Alignment seems straightforward when it is done well, but it can be tricky. Maggio et al. [2] noted room for improvement in the alignment between rationales and research questions in almost 65% of HPE scoping reviews. Echoing Levac et al. [3], they suspected that one reason for this misalignment is “rationales that are applicable broadly to a variety of knowledge synthesis methodologies and not necessarily specific to scoping reviews” (*p* 695). One strategy for testing your own alignment is to explain why you selected scoping review methodology: as Maggio et al. suggest, tell the reader “what factors influenced [the] decision to undertake a scoping review (e.g., the nature of the literature, the intricacies of the topic, the expertise of their research team, and/or their personal needs such as a graduate student familiarising herself with a topic)” (*p* 695). Another reason for misalignment is a research question that is too generic and not balanced by clearly articulated objectives that delimit the scope of the inquiry. For example, a generic question like “what is known about medical tourism” cannot support a strong search strategy: the scope needs to be tightened (e.g., medical tourism by Canadians for surgical procedures) in order to focus the work. Finally, even when you try to “balance” a broad question with focused objectives or sub-questions as Levac et al. have advised, be careful that your sub-questions still align with the rationale. If they feel more like detours than logical specifications of the overall focus, then you have a misalignment. For example, a sub-question about the ethics of medical tourism by Canadians for surgical procedures might feel misaligned if the rationale for the scoping review does not have any ethical flavour. Remember: alignment (or its lack) is judged by how well the rationale, question and sub-questions fit the overall story you’re laying out in the introduction—how coherently do they follow from your Problem, Gap and Hook?

A final note on alignment as it relates to the consultation exercise. The consultation is a unique strength of the scoping review. However, differing points of view have been expressed on its optional [20] or essential [3] nature, and it does not appear in the PRISMA-ScR [5] reporting guideline which can leave writers unsure about its role. As you frame the story for a review that included consultation, readers should see this step as aligned and necessary. For instance, you may anticipate a limited literature for scoping and thus seek insights from stakeholder consultation as primary data [15]. You may have scoped an ample literature but identified missing perspectives or voices that you explored using consultation [7]. Or you may use stakeholders to contextualize review findings in order to fully address the question being asked, as in Maggio et al.’s [2] consultation with “seven stakeholders to understand if and in what ways our findings resonated with their experiences conducting scoping reviews” (*p* 691). The consultation exercise will be described in your methods, but the introduction should set the reader up to expect it. For example, O’Brien et al.’s [16] rationale is to “advance policy and practice for people living with HIV” (*p* 449), which aligns well with their decision to consult with people with HIV to contextualize the literature and create a patient partner-informed research agenda.

## In summary

Every scoping review manuscript needs a *story* to frame the study. This Writer’s Craft offers strategies for telling this story in your introduction (Tab. 1). Use the Problem/Gap/Hook heuristic to establish why we need a synthesis in the first place. Structure the story so that it introduces setting and main characters, establishes that there is sufficient literature for a review, and acknowledges pre-existing reviews. Align the rationale for the work, the research question and the specific sub-questions or objectives—there should be no jarring detours from the storyline. Finally, keep it short: three or four paragraphs should suffice. Your introduction doesn’t need to detail the specifics; it should just sketch the curvature. And don’t worry about ‘giving away’ your results; your introduction is the story, not the synthesis.

**Table 1:** The story behind the synthesis: Strategies for an effective scoping review Introduction

1.	Characterize the gap: it is always a lack of synthesis, but of what sort?
2.	Say why the lack of synthesis matters—what problem does it create?
3.	Specify the value of a synthesis: what does it make possible? What does its absence threaten?
4.	Introduce setting & main characters via PCC framework, but beware extended inverted triangle
5.	Sketch the contours of existing literature to establish sufficient material to review
6.	Acknowledge previous reviews and distinguish this one from them
7.	Check alignment among rationale, research question and sub-questions
8.	Prepare the reader for the relevance of a consultation exercise
